# The Simplest Operation for Uncomplicated, Congenital Phymosis

**Published:** 1856-03

**Authors:** T. Furneaux Jordan


### *The Simplest Operation for Uncomplicated, Congenital Phymosis.*

By T. Furneaux Jordan, Esq., M. R. C. S.

Not only are Surgical authorities of opinion that circumcision is rarely, if ever, necessary; but those truly frightful slits, extending half-way up the penis, to be seen in the pretty engravings which adorn some (of our best, too) Surgical manuals are fast getting into chirurgical disfavor. The present mania, however, of attributing uncomplicated, congenital phymosis in every case to the unfortunate mucous lining of the prepuce alone, and the practice of heroically slitting up the same to the very point of its reflexion from the penis, has arisen rather from the hypothesis of theorists than from the enlightened experience of acute observers.

The non-dilatability of the congenitally phymosed prepuce is confined to the margin of the preputial orifice and to the skin and mucous membrane in its immediate vicinity; such non-dilatability undoubtedly extending to a greater distance on the inner than on the outer aspect of the foreskin.

The received opinion, touching the non-elasticity of the preputial lining in its entire extent, is so far from being correct, that ordinarily such lining, for some distance anterior to its point of reflexion, is arranged in rugous folds, like all other mucous membranes that are too large for the organ they line, save when the peculiar function of that organ is being exercised.

The opinion that the skin is not implicated in phymosed stricture, is equally incorrect. In one patient, on whom I operated with complete success, by far the tightest portion of the prepuce, after recovery from the operation, was the skin for two lines behind the cicatrices.

From the above remarks, it will be inferred that any incisions, which extend further than the parts forming the margin of the prepuce, and for a short additional distance on the mucous surface, are unnecessary, and hence cruel. A single incision, however, as described, would fail to secure the retraction of the prepuce, not because the incision is too limited, but because a single incision cannot possibly relieve the whole circumference of the congenitally-contracted preputial orifice; two, however, or at most three, of the small incisions in question would afford complete relief.

The mode of operating which I have adopted, and with signal success in its results, is this:—Having first induced local anæsthesia, by applying pounded ice to the penis for two minutes, I introduce one blade of a pair of scissors (blunt-pointed, yet cutting to the end) to the distance of ¼ an inch, between the glans penis and the prepuce, on one side of the penis, at a point midway between the frenum posteriorly, and the mesial line anteriorly. Both layers of the prepuce being divided to the extent mentioned, a similar incision is made at a similar point on the other side of the penis. The prepuce is now retracted to the extent allowed by the incisions, which by this proceeding are brought quite external, enclosing between their lips an uncut layer of lining membrane. This is divided on each side, by introducing one blade of the scissors, to the extent of, and immediately under, the orignal wound. The entire prepuce may then be retracted, a piece of wet lint wrapped round the penis, and the whole supported by a proper suspensory bandage. The patient need not lie in bed. Where three incisions seem preferable, they should be equidistant from each other, the third being at the mesial point anteriorly, the two lateral incisions should be a little nearer the frenum, than when two only are made.

The incisions may of course vary a line or two, one way or the other in extent, according as the constriction is more or less aggravated.

The recapitulatory points to which I would draw attention, are:—

1. That the skin is more, and
2. That the mucous membrane is less, involved, than is generally supposed.

3. That two, or at most three, comparatively small incisions will afford complete relief.
4. That no assistant is required, and
5. No instrument save a pair of scissors.
6. Two or three small incisions cause much less irritation, and heal much more quickly than one large one.
7. That the patient need not lie in bed.—*Medical Times and Gazette.*

